# Production of a toxic metabolite in 2,4-D-resistant GM crop plants

**DOI:** 10.1007/s13205-016-0387-9

**Published:** 2016-02-23

**Authors:** Paul F. Lurquin

**Affiliations:** 1 School of Molecular Biosciences, Washington State University, Pullman, WA 99164 USA; 2 c/o PO Box 369, Cannon Beach, OR 97110 USA

**Keywords:** Herbicide resistant crop plants, 2,4-dichlorophenol toxicity, 2,4-dichlorophenoxyacetic acid, 2,4-dichlorophenoxyacetic acid metabolites, Food safety

## Abstract

This Note questions the safety of crop plants engineered with transgenes coding for the degradation of the herbicide 2,4-dichlorophenoxyacetic acid (2,4-D) into its cytotoxic metabolite 2,4-dichlorophenol (2,4-DCP).

The herbicide 2,4-dichlorophenoxyacetic acid (2,4-D) was developed in the 1940s and used on a large scale for several decades. It has more recently been supplanted in agricultural markets by herbicides such as glyphosate and glufosinate. However, the appearance of weeds resistant to these herbicides has prompted biotech companies to engineer crop plants with transgenes conferring resistance to two herbicides, not just one. This would considerably delay the formation of herbicide-resistant mutant weeds. To that effect, Dow AgroSciences has recently released transgenic soybeans and corn resistant to both the herbicides dicamba or glyphosate and 2,4-D. These crops are described in layman’s terms in their website at http://www.enlist.com/en/how-it-works/enlist-traits. It should be noted that the author of this Note is not generally opposed to GM crop plants. Rather, he is of the opinion that scientific evidence should prevail in the release or rejection of such crops (see Lurquin 2001, 2002).

The idea of engineering 2,4-D resistance in plants is not new. A scenario to achieve this goal was presented by us in February 1985 at a symposium of the Weed Science Society of America in Seattle, Washington. This scenario was subsequently published in the peer-reviewed journal *Weed Science* (Perkins et al. 1987). Our reasoning was based on the ability of certain soil bacteria to degrade 2,4-D all the way to succinate (Fig. 1), a useful compound member of the Krebs cycle. To that effect we proposed to clone and transfer to plants one or more *tfd* genes from the soil bacterium known at the time as *Alcaligenes eutrophus*. This bacterial species is now known as *Cupriavidus necator*. Plasmid pJP4 harbored by *C. necator* 134 carries six genes designated *tfdA*–*F* that code for the degradative pathway of 2,4-D (Don and Pemberton 1985). The first gene of the pathway, *tfdA*, codes for an oxygenase that converts 2,4-D into 2,4-dichlorophenol (2,4-DCP) (Fig. 1). *C. necator* 134 grown in the presence of 2,4-D does indeed produce 2,4-DCP (Perkins et al. 1987). Interestingly, we first demonstrated that 2,4-DCP is much less toxic to plants than 2,4-D (Perkins et al. 1987). This observation was confirmed by others years later (Taylor et al. 1989). Thus, we reasoned that plants engineered with *C. necator’*s *tfdA* gene would convert phytotoxic 2,4-D into much less phytotoxic 2,4-DCP, hence rendering these plants resistant to the herbicide. This was indeed shown to be the case (Streber and Willmitzer 1989; Lyon et al. 1989; Bayley et al. 1992). Dow Agrosciences’ recently released 2,4-D resistant transgenic corn and soybeans were engineered with *C. necator’s* *tfdA* homologs isolated from *Sphingobium herbicidivorans* and *Delftia acidovorans* and thus produce 2,4-DCP (Wright et al. 2010).

**Fig. 1:**
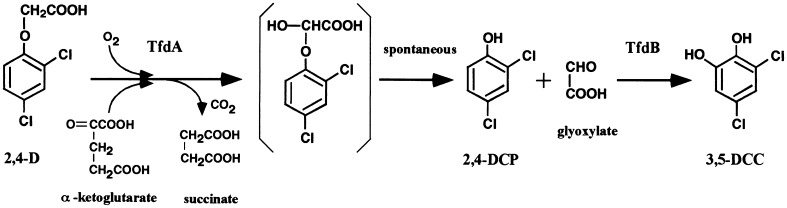
Partial catabolism of 2,4-dichlorophenoxyacetic acid (2,4-D) by the two enzymes coded for by *tfdA* and *tfdB*. *2,4-DCP* 2,4-dichlorophenol; *3,5-DCC* 3,5-dichlorocatechol. *TfdA* (the product of the *tfdA* gene) converts phytotoxic 2,4-D into much less phytotoxic 2,4-DCP (Perkins et al. 1987). The latter is toxic to a variety of animal cells, however (see text). *TfdB* converts 2,4-DCP into 3,5-DCC, a compound that still displays phytotoxicity (Liao et al. 2006). Tobacco plants transgenic for *tfdB* exist (Perkins et al. 1990a, b) but have not been used in phytotoxicity studies. Human toxicity of 3,5-DCC is present but information is scant. According to its Material Safety Data Sheet (MSDS) 3,5-DCC can cause eye, skin and respiratory irritation. The remaining phytotoxicity of 3,5-DCC can be reduced by inserting the third gene of the pathway, *tfdC* (Liao et al. 2006). *TfdC* converts 3,5-DCC into dichloro cis, cis-muconate by opening the aromatic ring (not shown). The human toxicity of dichloro cis, cis-muconate is unknown. Introducing the whole 2,4-D degradation pathway into plants would allow them to convert 2,4-D into succinate and acetyl coA (Perkins et al. 1990a)

Unfortunately, much reduced phytotoxicity does not necessarily mean that *tfdA*-(or analogous) containing 2,4-D resistant crop plants are safe for consumption. Indeed, 2,4-DCP is cytotoxic to a variety of animals and animal cell lines. The International Agency for Research on Cancer (IARC), a branch of the World Health Organization, has categorized 2,4-DCP as a compound belonging to group 2B, that is, “a possible carcinogen based on inadequate evidence in humans and limited evidence in experimental animals.” But in fact, the IARC in 2015 also designated 2,4-D as a group 2B compound (http://www.iarc.fr/en/media-centre/pr/2015/pdfs/pr236E.pdf). However, a meta-analysis of the literature (Goodman et al. 2015) does not support an association between 2,4-D and non-Hodgkin’s lymphoma, gastric cancer, or prostate cancer, as proposed earlier by some. Such meta-analysis does not seem to exist for 2,4-DCP and cancer risk. Nevertheless, extensive reports reviewing the earlier literature up to about the year 2000 and published by the IARC (http://monographs.iarc.fr/ENG/Monographs/vol71/mono71-34.pdf) and the Japanese Ministry of Economy, Trade and Industry (http://www.meti.go.jp/english/report/downloadfiles/gED0307e.pdf) both agree that 2,4-DCP presents low acute toxicity when delivered orally in mice and rats. For example, in rats, no effect on survival is seen after 13 weeks on 2000 mg/kg/day. In mice, no mortality is observed after 13 weeks on 2600 mg/kg/day (Syracuse Research Corporation 1992; also see Gorzinski et al. 1987). In addition, both reports indicate the absence of evidence linking 2,4-DCP and tumorigenesis in experimental animals. Further, 2,4-DCP registers negative in the Ames test but scores positive in the sister chromatid exchange test in CHO cells in vitro (see the National Toxicology Program 1989). 2,4-DCP also scores positive in the aneuploidy test with Chinese hamster V79 lung cells in vitro and induces mutations in the *tk* gene in mouse lymphoma L5178Y cells also in vitro. (see the IARC report for references to all these observations).

More recently, 2,4-DCP has been linked to a variety of negative metabolic and additional genotoxic effects. For example, 2,4-DCP injected intraperitoneally into Swiss mice at one half the LD50 (180 mg/kg) displayed chromosome aberrations in bone-marrow and spermatocyte cells. 2,4-DCP also induced sperm head abnormalities in these animals (Amer and Aly 2001). Further, 2,4-DCP induces apoptosis in L929 mouse cells (Chen et al. 2004) and grass carp primary hepatocytes (Li et al. 2013), inhibits antioxidant enzyme activities in human erythrocytes in vitro (Bukowska 2003), exhibits androgenic effects potentiated by dihydrotestosterone in human prostate cancer cells (Kim et al. 2005) and interferes with the transcription of genes involved in steroidogenesis in H295R human adrenocortical carcinoma cells in vitro (Ma et al. 2012). Ma et al. (2012) also observed disruption of endocrine function in zebrafish in vivo. Also, negative reproductive effects were observed in a two-generation study in 2,4-DCP-treated Wistar-Hannover rats (Aoyama et al. 2005). Another report shows that 2,4-DCP induces global DNA hypermethylation in the liver of goldfish (Zhang et al. 2014). The use of fish species in some of the studies is not surprising as 2,4-DCP is a pollutant found in industrial effluents.

In view of some of the noxious properties displayed by 2,4-DCP already known over a decade ago, serious concerns regarding the safety of 2,4-D resistant crops producing 2,4-DCP were voiced earlier (Lurquin 2001, 2002). To try to circumvent the toxicity of 2,4-DCP, we advocated the further engineering of 2,4-D resistant plants with downstream catabolic genes, such as *tfdB* and beyond (Lurquin 2002). To that effect, we have cloned and sequenced the *tfdB* and *tfdC*–*F* operons from pJP4 (Perkins et al. 1988, 1990a). For example, Fig. 1 shows that the product of *tfdB* converts 2,4-DCP into 3,5-dichlorocatechol (3,5-DCC) which *may* be less toxic than 2,4-DCP. However, even this step does not totally solve the problem of 2,4-DCP’s permanence in resistant plants. Indeed, it has been shown (Perkins et al. 1990b) that tobacco plants transgenic for *C. necator’s* *tfdB* fail to convert all their fed 2,4-DCP into 3,5-DCC, possibly as a result of 2,4-DCP’s affinity for cellular structures and/or metabolization that limit access to the *tfdB* gene product. In fact, Laurent et al. (2007) demonstrated that non-transgenic tobacco cells in suspension culture incubated with radiolabeled 2,4-DCP form glycoside and glucoside conjugates of the latter. This, write these authors, might be problematic, as these conjugates, when consumed by animals and humans, may release 2,4-DCP in hydrolysis reactions in their digestive tracts.

In conclusion, the safety of 2,4-D resistant crop plants remains a controversy. It behooves biotech companies responsible for the release of 2,4-D resistant crops, as well as federal agencies, to fully disclose to the public the results of extensive toxicology studies with these crops. Unfortunately, Wright et al. (2010), the creators of 2,4-D resistant corn and soybeans, do not discuss the accumulation and fate of 2,4-DCP in their article and neither does Dow AgroSciences’ relevant website (http://www.enlist.com/en/how-it-works/enlist-traits).

As mentioned above, the full degradative pathway of 2,4-D has been cloned and sequenced (Perkins et al. 1990a). Thus, nothing prevents biotechnologists from transferring several *tfd* genes into target crop plants to make them safer for human consumption. As a first step in that direction, Liao et al. (2006) have shown that plants transgenic for *tfdC* can detoxify 3,5-DCC, the second metabolite in the 2,4-D degradative pathway (Fig. 1), by converting it to dichloro cis, cis muconate. They also showed that *Arabidopsis thaliana* plants possess *tfdD* and *tfdE* orthologs whose products are seemingly able to further catabolize cis, cis muconate. It is not known whether 2,4-D resistant GM corn and soybeans harbor such orthologs. Thus, biotech companies should take advantage of transferring several *tfd* genes (or analogs) into target crop plants instead of releasing potentially harmful GM crops prematurely.
